# Simultaneous study of antioxidant activity, DNA protection and anti-inflammatory effect of *Vernonia amygdalina* leaves extracts

**DOI:** 10.1371/journal.pone.0235717

**Published:** 2020-07-13

**Authors:** Wei-Te Wang, Su-Fen Liao, Zih-Ling Wu, Chia-Wei Chang, Jane-Yii Wu

**Affiliations:** 1 Department of Food Science and Biotechnology, Da-Yeh University, Changhua, Taiwan; 2 Department of Physical Medicine and Rehabilitation, Changhua Christian Hospital, Changhua, Taiwan; National University Singapore Yong Loo Lin School of Medicine, SINGAPORE

## Abstract

*Vernonia amygdalina* (VA) has been reported to have antioxidant potential; however, its DNA protection and anti-inflammatory properties remain unclear. We aimed to investigate whether aqueous (WEVAL) and alcoholic (EEVAL) VA extracts exert similar antioxidant, DNA protection and anti-inflammatory effects and attempted to explore the mechanism underlying the anti-inflammatory effects. These results demonstrated that WEVAL had greater polyphenolic and flavonoid contents, as well as a stronger reducing power, DPPH radical scavenging and DNA protective activity. Moreover, both extracts reduced lipopolysaccharide (LPS)-induced expression of COX-II, iNOS, pro-inflammatory factors, including NO, TNF-α, IL-1β, and IL-10. Compared with WEVAL, EEVAL was a more potent inflammatory inhibitor. Both extracts similarly inhibited LPS-induced MAPK (p38) and NF-κB expression. Our findings indicate that WEVAL and EEVAL have diverse antioxidant and anti-inflammatory effects. WEVAL had a stronger antioxidant and DNA protection activity; contrastingly, EEVAL had a stronger anti-inflammatory ability. The anti-inflammatory activity involves reduced pro-inflammatory cytokines through NF-κB down-regulation and MAPK inhibition. These results demonstrated that production of WEVAL and EEVAL from VA leaves may provide a safe and efficacious source of pharmaceutical applications, with antioxidant, DNA protective and anti-inflammation activities.

## 1. Introduction

Inflammation is a self-protective body mechanism for the prevention and removal of harmful stimuli. Immune cells, especially macrophages, play an important role in the inflammation process. Through lipopolysaccharide (LPS) stimulation, macrophages initiate intracellular signal cascades for the synthesis of the pro-inflammatory cytokine, e.g., IL-1, IL-6, and TNF-α [1]. The most important intracellular signaling proteins for inflammation are NF-kappa B (NF-κB) and mitogen-activated protein kinases (MAPKs). Inducible nitric oxide synthase (iNOS) and cyclooxygenase II (COX-II) are pro-inflammatory proteins that induce the production of secondary mediators, including nitrite (NO) and prostaglandin, to enhance the inflammation process.

Excess radical accumulation results in oxidative pressure, which are harmful to human health. Reactive oxygen species (ROS) are major free radicals in the human body and causes oxidative injury of cell and DNA and induce human disease like cancer [2, 3]. By acting as ROS or free radical scavengers, antioxidants can directly decrease the oxidative pressure damages [4]. Therefore, appropriate inflammation regulation and antioxidant activity promotion are important.

*Vernonia amygdalina* (VA) belongs to the Asteraceae family and grows widely in Africa. Its leaves are used in African folk medicine. VA leaves contain several bioactive phytochemicals, including flavonoids, phenolic acid, terpenes, and coumarins. Many studies have indicated that VA has some medicinal potential, including antioxidant, antibiotic and anti-cancer [5, 6]. Studies on the antioxidant effects of VA have used both aqueous and alcoholic extracts. However, there have been only a few studies comparing the two extracts; further, the results of those studies are controversial [5]. Polyphenols and flavonoids are high correlation with the antioxidant and anti-inflammatory activity of plants. Luteolin is a flavonoid in VA that has been reported to have strong antioxidant activity [7]. Further, luteolin also has been reported to prevent pro-inflammatory cytokine production [8]. The antioxidant activity of VA leaves is highly correlated with polyphenol and flavonoid levels; however, differences in the antioxidant capacity and polyphenol content between aqueous extracts of VA leaves (WEVAL) and alcoholic extracts of VA leaves (EEVAL) remain unclear. Moreover, only one animal study has been conducted, which reported that WEVAL could relieve croton oil-induced rat ear inflammation [9]. There has been no biochemical study on the anti-inflammatory effects of VA.

In this study, we aimed to investigate the effects of WEVAL and EEVAL on antioxidant, DNA protection and LPS-induced inflammation and to determine the underlying biochemical mechanism. Moreover, we aimed to compare the antioxidant and anti-inflammatory effects between the two extracts and to clarify whether polyphenols and flavonoids were the main anti-inflammatory VA components.

## 2. Materials and methods

### 2.1. Materials (Plant and chemicals)

The plant of *Vernonia amygdalina* (VA) was bought from a plant farm in Tanwei, Changhua, Taiwan.

Gallic acid, sodium nitrite, aluminum chloride (AlCl_3_), sodium hydroxide (NaOH), sodium nitrite (NaNO2), sodium carbonate (Na_2_CO_3_), folinciocalteu reagent, quercetin, phosphate-buffered saline (PBS), 3-[4,5-dimethylthiazol-2-yl]-2,5- diphenyltetrazolium bromide (MTT), dimethyl sulfoxide (DMSO), and 1,1-diphenyl- 2-picryl- hydrazyl (DPPH), were obtained from Merck Co. (Darmstadt, Germany). All the chemicals and solvents used in the study were of analytical grade. Enzyme-linked immunosorbent assay (ELISA) kits for IL-1β, IL-6, IL-10, and TNF-α) were obtained from Ebioscience, Inc (San Diego, USA). Primary antibodies for detecting NF-κB p65, phospho-NF-κB p65, iNOS, and COX-II were obtained from Cell Signalling Technology (Beverly, MA, USA). Secondary antibody for phospho-NF-κB p65 in immunofluorescence staining was obtained from Sigma-Aldrich (St. Louis, MO, USA). MAPKs and secondary antibodies were obtained from Signalway Antibody (College Park, MD, USA) and GeneTex, Inc (Irvine, CA, USA), respectively. 2.2. Preparation of WEVAL and EEVAL sample.

Leaves from a 6-month-old VA were air-dried. Using a stainless-steel grinder, and the leaves were then ground into a fine powder (less than < 10 mesh) and were then stored at room temperature. Next, 10 g of dried VA leaf powder were extracted using distilled water in the autoclave for 1 h (WEVAL sample) and 70% ethanol in an ultrasound sonicator for 1 h (EEVAL sample), respectively. This process was repeated three times. Using vacuum, the extract was filtered and then, using a rotary evaporator, dry extract was obtained. The WEVAL and EEVAL extract were finally evaporated to obtain a final weight, respectively.

### 2.3. Cell culture

RAW 264.7 cells was obtained from the Bioresource Collection and Research Center, Food Industry Research and Development Institute (Hsinchu, Taiwan), and cultured in Dulbecco`s Modified Eagle Media supplemented with 10% fetal bovine serum and 1% penicillin-streptomycin in an incubator at 37 °C, 5% CO_2_, and 95% humidity.

### 2.4. Cell viability

The RAW 264.7 cells were cultured at a density of 2 × 10^6^ cells/well in 96-well plates and pretreated with the VA leaves extracts for 24 h. After washing with PBS, we then added MTT and incubated for 3 hours. After mixing with 10% DMSO, we measured absorbance using an ELISA reader at a 570-nm wavelength.

### 2.5. Total phenolic and flavonoids content

The total phenolic content of the extracts were measured using the Folin-Ciocalteu assay as previously described [10]. We added WEVAL and EEVAL into separate test tubes and mixed with 100 μL of Folin-Ciocalteu’s reagent for 3 min followed by sodium bicarbonate for 30 min. We measured the absorbance at 735 nm and compared it with a standard compound, gallic acid. The results were expressed as gallic acid equivalents (GAE) in mg/g.

The total flavonoid content of the extracts were determined using the aluminum chloride colorimetric method [11]. WEVAL and EEVAL diluted using distilled water was added to a test tube and mixed with 0.3mL of 5% sodium nitrite (NaNO_2_) solution for 6 min followed by 0.3 mL of 10% aluminum chloride (AlCl_3_) solution. Finally, after a 5-min reaction, 1mol/L NaOH was added to the mixture and the absorbance was measured at 510 nm. We used quercetin as the standard compound and calculated the total flavonoid content using the calibration curve for quercetin. The results were expressed as mg quercetin equivalents per mL extract.

### 2.6. DPPH free radical scavenging assay

As previously described, using DPPH assay, radical scavenging activities of the VA leaves extracts were determined [12], with some modification. We prepared different WEVAL and EEVAL dilutions and added 150 μL of DPPH (0.2 g/100 mL), which was left to stand for 30 min. We measured the absorbance at 517 nm using ELISA reader with BHT as the positive control. The inhibition ration was calculated as follows:

$$\text{The}\;\text{inhibition}\;\text{ratio}\;(\text{\%})=\frac{\text{absorbance}\;\text{values}\;\text{of}\;\text{the}\;\text{extract}\;\text{sample}}{\text{absorbance}\;\text{values}\;\text{of}\;\text{the}\;\text{blank}\;\text{sample}}\times 100$$

### 2.7. Reducing power assay

The reducing power of the extracts were determined by monitoring the reduction of ferric iron to ferrous ion as previously described [13]. We mixed different WEVAL and EEVAL dilutions with 50 μL phosphate buffer (pH 6.6, 200 mM) and 50 μL of potassium ferricyanide (1%, w/v) at 50 °C for 20 min. Next, we added 50 μL trichloroacetic acid (10%, w/v) followed by centrifugation at 9000 rpm for 3 min. Finally, 50 μL supernatant solutions were diluted to double volume using distilled water and mixed with 50 μL ferric chloride dissolved in water (0.1%, w/v). After a 10-min reaction time, we measured the absorbance at 700 nm compared with a blank. Positive controls used were butylated hydroxytoluene (BHT) and α-tocopherol. Increased absorbance of the extract indicated a higher reducing power.

### 2.8. DNA strand protection assays

The protection activity of oxidative DNA damage of WEVAL were determined by the DNA nicking assays [14], which assessed the DNA strand break according to the ratio of circular supercoiled pCI neo plasmid DNA converted into nicked circular or further degraded forms. In brief, reaction mixtures contained 2.5μL of supercoiled plasmid DNA (150 ng/μL), 10μL of a Fenton reagent (30 mM hydrogen peroxide, 100 μM ferric chloride and 100 μM ascorbic acid) and 5μL of WEVAL or quercetin (250 μg /mL), finally add distilled water to a total volume of 20μL. Reaction mixtures were incubated at 37 °C for 30 min and plasmid DNA forms were separated on 0.7% agarose gels. Nucleic acid stain of plasmid DNA were used SafeView (Applied Biological Materials (ABM) Inc., Richmond, Canada).

To assessed antioxidant activities of the extracts, amount of supercoiled and nicked forms plasm DNA on agarose gels was quantified by AlphaImager Mini (proteinsimple) instrument and band intensities was by Gelpro software. Supercoiled plasmid DNA was incubated alone as negative control and with the Fenton reagent mixture as positive control. Data are the ratio of supercoiled DNA amount relative to that in negative controls and expressed as a percentage. Protective activities of the samples were calculated from amount of supercoiled and nicked plasmid DNA using the following equations [15]:

$$Protection\;of\;supercoiled\;plasmid\;(\%)\;=\;\frac{supercoiled\;form\;intensity}{pCI\;neo\;DNA\;band\;intensity}\;x\;100$$

$$Protection\;of\;nicked\;plasmid\;(\%)\;=\;\frac{nicked\;form\;intensity}{pCI\;neo\;DNA\;band\;intensity}\;x\;100$$

### 2.9. Nitrite determination

The nitrite were determined using a Griess reaction test. We plated 1 mL of RAW 264.7 cells in a 6-well plate. The cells were pretreated with the VA leaf extracts for 1 h and then 1 μg/mL LPS was added. After 24-hour incubation, we isolated the supernatants. Next, we mixed 100 μL of cell supernatants with Griess reagent of equal volume at room temperature for 10 min. We measured absorbance at 540 nm and compared it with that of known sodium nitrite concentrations to calculate the nitrite concentration.

### 2.10. Enzyme-linked immunosorbent assay

RAW 264.7 cells were pre-cultured at a density of 1 × 10^5^ cells/well in 24-well plates for 24 h and then treated with 1 μg/mL LPS and 100 μL of the extracts for another 24 h. We placed the samples in 96-well plates and quantified the The concentration of IL-1β, IL-6, IL-10 and TNF-α released into the medium was quantified using the ELISA kits (Ebioscience, San Diego, USA) as per the manufactures’s instructions. We measured the absorbance at 450 nm~540 nm and calculated the absolute concentration using the standard curve.

### 2.11. Western blot analysis

The RAW 264.7 cells at a density of 2 × 10^5^ cells/well were planted in 6-well plates for 24 h. After treatment with each extract for 1 hour, we cultured the cells with 2 μg/mL LPS for another 24 h. The treated cells were mixed with protein inhibitor cocktail and RIPA buffer for cell lysis and then centrifuged at 12000 rpm at 4 °C for 3 min to collect cellular protein. The extracted cellular protein was separated using SDS-PAGE and transferred to polyvinylidene difluoride (PVDF) membranes. The membranes were blocked using 5% skimmed milk. The membrane was incubated overnight at 4 °C with a 1:1000 dilution of specific primary antibodies for detecting NF-κB p65, phospho-NF-κB p65, iNOS, and COX-II (Cell Signalling Technology, Denver, USA) and MAPKs (Signalway Antibody, Baltimore, USA). Next, the membrane was incubated with a 1:5000 dilution of secondary antibody (GeneTex, Irvine, USA) for 1 h and placed in an enhanced solution for chemiluminescence detection (Sage creation, Beijing, China). Chemiluminescence analysis was conducted using AlphaEaseFC software.

### 2.12. NF- ĸB nuclear translocation analysis

The nuclear translocation of NF-κB subunit p65 was analyzed for the activation of NF-κB in macrophages. After treatment with each extract, cells were washed with PBS and stained with DAPI for 15 min, followed by fixed with 4% paraformaldehyde for15 min at room temperature. Block specimen in Blocking buffer (X PBS / 5% normal serum / 0.3% Triton^™^ X-100) for 1 h and incubated with rabbit anti-p65 primary antibody (1:100, Cell Signalling Technology, Denver, USA) for overnight at 4 °C. Then the cells were washed with PBS and incubated with Anti-Rabbit IgG FITC-antibody (1:80, Sigma-Aldrich, MO, USA) for 1–2 hr at room temperature in the dark. Cells were rinsed with PBS and covered with antifade reagent. Finally, nuclear translocation of NF-κB subunit p65 protein was assessed under fluorescent microscope (OLYMPUS CKX41, Japan). To determine the subcellular regions of protein co-localization, the same field images were merged using with OLYMPUS cellSens software.

### 2.13. Statistical analysis

Descriptive statistics were expressed as mean and standard deviation (SD) from three independent experiments. Differences between treatments were identified using Student’s t-test or ANOVA followed by Dunnett’s test. Statistically signifcant changes were classifed as signifcant (*) where p < 0.05, more signifcant (**) where p < 0.01 and highly significant (***) where p < 0.001 as compared with background group.

## 3. Results and discussion

### 3.1. Total phenolic and flavonoid levels and antioxidant activity of WEVAL and EEVAL

Polyphenol and flavonoid are two nature phytochemicals which have been reported to be highly correlated to antioxidant and anti-inflammatory ability of plants. Flavonoids belong to a subgroup of polyphenols and have variable phenolic structures. Luteolin, a flavonoid found in VA, has been reported to be a strong antioxidant[7] and decrease the production of pro-inflammatory cytokines[8]. High phenolic and flavonoid contents could play a significant role in the antioxidant ability of VA leaves. The VA leaves underwent extraction using different solvent systems (aqueous and alcoholic). Tables 1 and 2 show the total phenolic and flavonoid levels in WEVAL and EEVAL. The highest phenolic and flavonoid levels in WEVAL were 0.327 mg GAE/g and 3.393 mg QE/g, respectively, while the corresponding values for EEVAL were 0.011 mg GAE/g and 0.347 mg QE/g, respectively. WEVAL contained higher polyphenol and flavonoid contents compared to those in EEVAL.

**Table 1:** Total phenolic contents, flavonoid contents and DPPH radical-scavenging activity of WEVAL and EEVAL.

Sample. (mg/mL)	Total polyphenolic content as gallic acid equivalent (mg/g)	Total flavonoid content as quercetin equivalent (mg/mL)	DPPH radical-scavenging activity (% of Control)
WEVAL			
0.006	—	—	10.297±1.880
0.012	0.011±0.002	0.003±0.001	11.448±0.561
0.06	0.037±0.003	0.280±0.002	41.611±0.421
0.12	0.069±0.003	0.626±0.004	81.397±0.084
0.3	0.166±0.004	1.663±0.003	89.955±0.168
0.6	0.327±0.004	3.393±0.003	89.030±0.365
EEVAL			
0.014	—	—	—
0.028	—	—	4.006±1.901
0.142	0.002±0.001	0.025±0.001	3.889±0.848
0.284	0.003±0.001	0.061±0.006	5.702±2.778
0.711	0.006±0.001	0.168±0.005	13.772±1.316
1.423	0.011±0.001	0.347±0.007	27.310±0.702

Values are mean of pooled samples analysed in triplicate ± SD;

“—” is not detected.

**Table 2:** Total phenolic, total flavonoid contents and reducing power of WEVAL and EEVAL.

Sample (mg/mL)	Total polyphenolic content as gallic acid equivalent (mg/g)	Total flavonoid content as quercetin equivalent (mg/mL)	Reducing power (OD_700_)
WEVAL			
0.0015	—	—	0.016±0.003
0.003	—	—	0.033±0.004
0.015	0.0124±0.001	0.021±0.001	0.146±0.009
0.03	0.020±0.002	0.107±0.001	0.294±0.001
0.075	0.045±0.003	0.367±0.003	0.681±0.008
0.15	0.085±0.004	0.799±0.003	1.058±0.014
EEVAL			
0.0035	—	—	—
0.0071	—	—	0.009±0.003
0.036	—	—	0.012±0.001
0.071	0.002±0.001	0.007±0.001	0.031±0.001
0.178	0.003±0.001	0.034±0.002	0.045±0.001
0.356	0.004±0.001	0.079±0.004	0.064±0.005

Values are mean of pooled samples analysed in triplicate ± SD;

“—” is not detected.

### 3.2. Antioxidant activity of WEVAL and EEVAL

The antioxidant activities of WEVAL and EEVAL were determented using the DPPH radical scavenging and reducing power tests. DPPH is a relatively stable organic radical whose absorbance at 517 nm decreases after accepting an electron or hydrogen radical. Assessing the DPPH radical scavenging activity is a frequently used means of determining the antioxidant activity [13]. We calculated the extract- and BHT-induced percentage decrease in the DPPH absorbance to determine the extracts’ antioxidant activity. Table 1 shows the radical scavenging ability of WEVAL and EEVAL. There was a gradual increase in the antioxidant activity of WEVAL and EEVAL with increasing concentration; further, WEVAL had higher antioxidant activity than EEVAL. The WEVAL scavenging effect nearly matched that of BHT when the WEVAL concentration was > 0.3 mg/mL. EEVAL did not show radical scavenging activity at a low concentration and only achieved 27.3% activity when its concentration was 1.423 mg/mL.

Reducing power is generally associated with the presence of reductones, e.g., the hydroxyl group in phenolic compounds [16] and strongly associated with plant antioxidant activity and is considered as a significant antioxidant ability indicator [17]. A higher extract absorbance indicates a higher antioxidant activity. As shown in Table 2, there was a dose-dependent increase in the reducing powers of both WEVAL and EEVAL. The reducing powers of WEVAL at 0.15 mg/mL and EEVAL at 0.36 mg/mL were 1.058 and 0.064, respectively. WEVAL had a higher reducing power than that of EEVAL. Our reducing power test results were similar to those of the DPPH radical scavenging assay with both indicating that WEVAL had a stronger antioxidant ability.

Previous findings are unclear on whether WEVAL and EEVAL have a stronger antioxidant effect [5]. A previous study reported that EEVAL had a higher antioxidant effect [6]. Contrastingly, Atangwho et al. reported that WEVAL had the best antioxidant potential [18], which is consistent with our findings. Our findings showed that the greater phenolic and flavonoid content in WEVAL allowed it a greater antioxidant ability.

### 3.3. DNA protection activity of WEVAL

**Fig 1:**
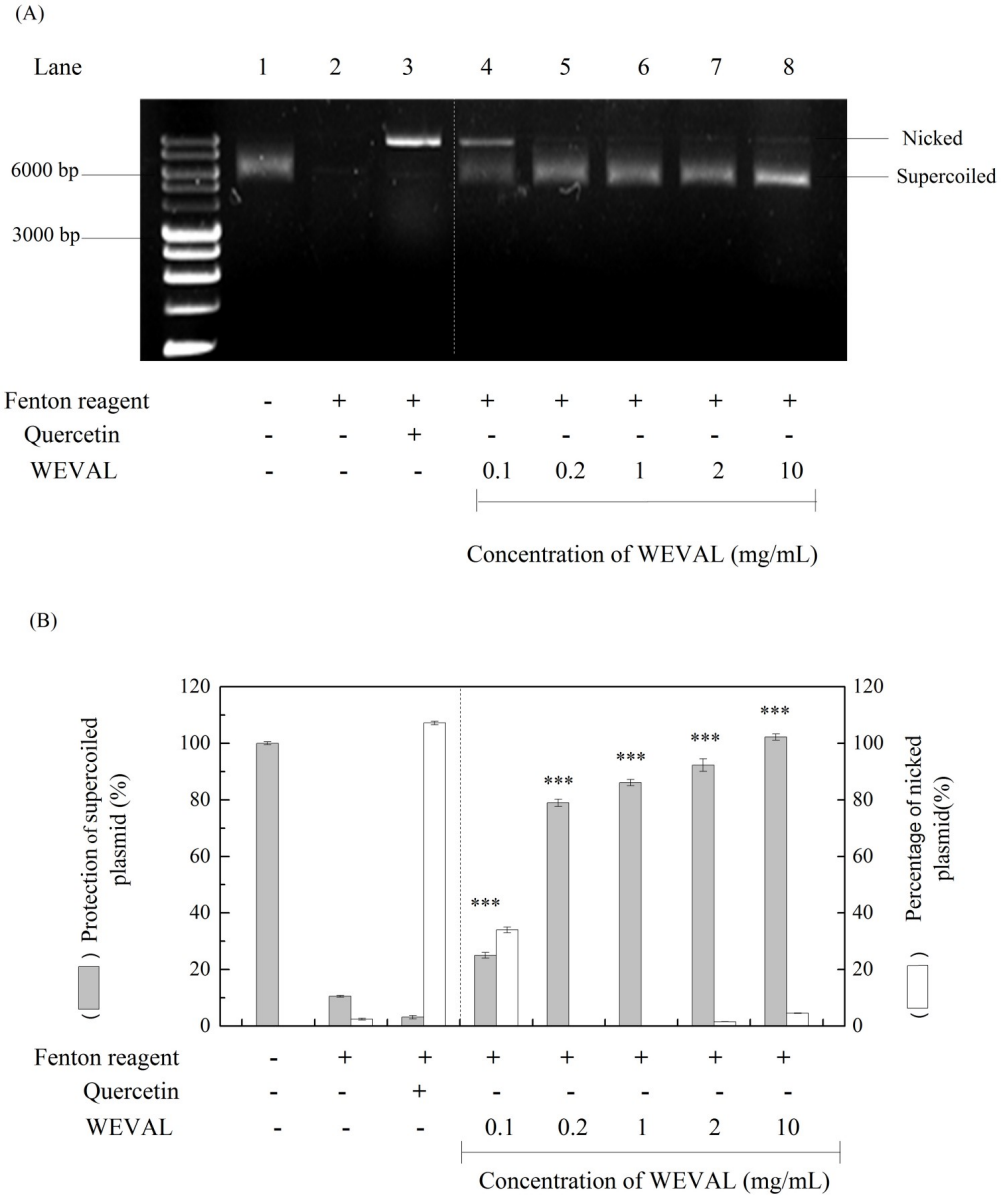
Effects of WEVAL in pCI neo DNA protection assays. (A) agarose gel of pCI neo DNA after exposure to Fenton reagents and protective treatments; (B) percentage quantification of supercoiled and nicked pCI neo forms; Lane 1, pCI neo DNA, negative control; Lane2, pCI neo + Fenton reagents, negative control; Lane3, pCI neo + Fenton reagent + 250 μg/mL quercetin, positive control; Lanes 4–8, pCI neo + Fenton reagent + various concentrations(lane 4–0.1, lane 5: 0.2, lane 6: 1, lane7: 2, lane 8: 10 mg/mL) of WEVAL. Statistically significant changes were clustered as highly significant (***) where p < 0.001 as compared with Fenton reagent group.

ROS can damage DNA and cause cell injury and human disease like cancer. Bedsides DPPH free radical scavenging ability and reducing power which determine WEVAL had a stronger antioxidant activity, WEVAL protection activities of DNA damage from antioxidant were also need to investigate. DNA nicking assays offer an in-vitro model to sensitively determine the production of DNA damaging radicals [19] and employ Fenton reactants to produce hydroxyl radicals that cause supercoiled DNA strand break, then converted it into nicked circular form [20]. By their relative electrophoretic mobility rates on gel, supercoiled and nicked plasmid forms were distinguished directly. Thus, DNA nicking assays were used to determine the DNA protection activity of VA through checking WEVAL against DNA strand break by hydroxyl radical species[21]. As presented in Fig 1(A), WEVAL reduced DNA damage markedly at concentrations of 0.1–10 mg/mL, with 25–100% protection of the supercoiled form, respectively (Fig 1(B), lanes 4–8). Unlike WEVAL protecting double strand DNA, quercetin effectively protected single-stranded nicked circular from oxidative fragmentation [22]. Our data showed WEVAL had protective effects on oxidative DNA damage and these effects are likely related to the presence of polyphenol and flavonoid compounds.

### 3.4. Effect of WEVAL and EEVAL on RAW 264.7 cell viability

**Fig 2:**
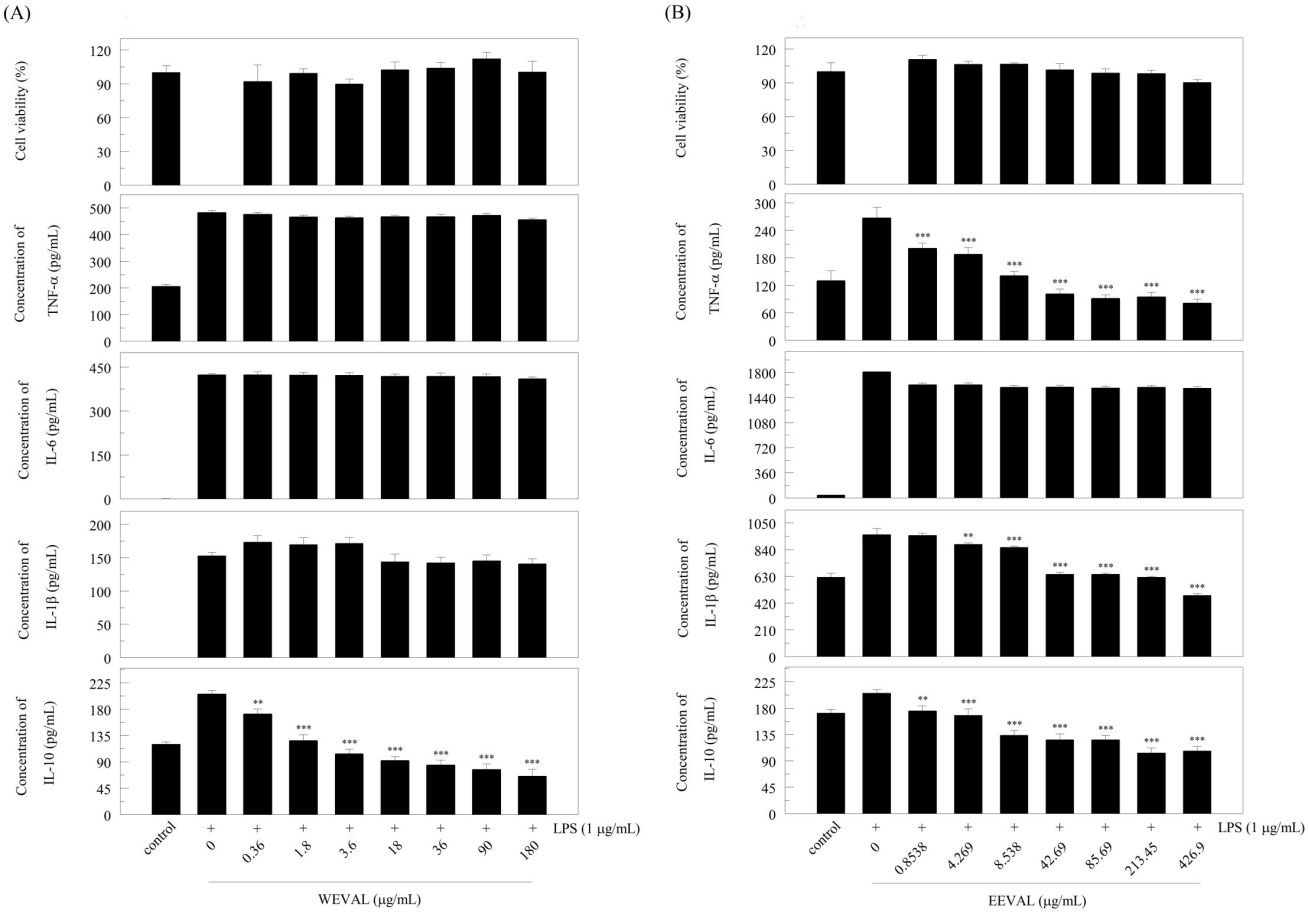
Effects of the VA leaf extracts on RAW 264.7 cell viability and on cytokine production in LPS-stimulated macrophages. Effect of (A) WEVAL and (B) EEVAL on cell viability and TNF-α, IL-6, IL-1β, and IL-10 production. Results are expressed as mean ± SD of triplicate experiments. Statistically significant changes were clustered as more significant (**) where p < 0.01 and highly significant (***) where p < 0.001 as compared with LPS-stimulated group.

We used the MTT assay to evaluate the cytotoxic effect of WEVAL and EEVAL. First, we investigated the cytotoxicity of various WEVAL and EEVAL concentrations. The results are presented in Fig 2(A) and 2(B). WEVAL at 180 μg/mL or EEVAL at 426.9 μg/mL had no effect on RAW 264.7 cell viability. These findings suggest that both WEVAL and EEVAL were non-cytotoxic at concentrations of 180 μg/mL and 426.9 μg/mL, respectively. The subsequent cell experiments were conducted with VA leaf extracts with concentrations below the aforementioned non-cytotoxic concentrations.

### 3.5. WEVAL and EEVAL inhibited pro-inflammatory cytokine expression

TNF-α, IL-1β, and IL-6 are potent pro-inflammatory factors that regulate the release of many inflammatory cytokines and immune cell activation [23]. TNF-α is primarily produced by ligand-stimulated activated macrophages as a host defense response to microbes. TNF-α mediates the cell apoptosis cascade and is involved in various pathophysiological events, including septic shock and inflammatory disease (Ceramil & Beutler, 1988; Rath & Aggarwal, 1999). IL-1β plays an important homeostatic role in maintaining and regulating normal physiological functions. IL-1β overproduction causes numerous pathological events, including arthritis and vascular diseases [24]. Fig 2(A) and 2(B) demonstrate that all EEVAL concentrations significantly reduced LPS-induced TNF-α production; however, only WEVAL with concentration > 180 μg/mL achieved this effect. Similarly, EEVAL showed a more significant reduction in IL-1β expression.

IL-6 plays a vital role in the host defense and lymphocyte differentiation. IL-6 dysregulation contributes to several chronic and autoimmune diseases[25]. Only EEVAL had a weak inhibitory effect on LPS-induced IL-6 production. Although the VA leaf extracts showed weak inhibition of IL-6 expression, they were potent TNF-α and IL-1β inhibitors.

IL-10 is a major immunoregulatory factor with its primary function being inhibition of cytokine synthesis [26]. In macrophages, IL-10 inhibits the production of TNF-α, IL-1, and IL-6 [27] and modulates NO synthesis by inhibiting Th cells [28]. Both WEVAL and EEVAL showed IL-10 inhibitory effects; however, EEVAL showed weaker IL-10 inhibition and a stronger anti-inflammatory ability compared to WEVAL.

These findings indicate that both WEVAL and EEVAL inhibited TNF-α, IL-1β, and IL-10. They suggest that WEVAL and EEVAL could attenuate LPS-induced inflammatory activity through macrophage activation. Compared with WEVAL, EEVAL had a stronger inhibitory effect on TNF-α and IL-1β production but a relatively weaker inhibitory effect on IL-10 production. Further, EEVAL had a stronger anti-inflammatory effect on LPS-activated RAW 264.7 macrophages.

### 3.6. WEVAL and EEVAL inhibited iNOs and NO production

A free radical, NO plays an important role in inflammation regulation. Dysregulation of macrophage production of NO might play a critical role in human inflammatory diseases [29]. At the inflammation site, macrophages transcribe iNOS upon pro-inflammatory cytokine and LPS exposure. Further, TNF-α plays a key role in iNOS induction and NO synthesis [30]. Moreover, IL-1β can increase the gene expression and synthesis of COX-II and iNOs [24]. Cytokines such as IL-10 can also modulate NO synthesis through Th cell inhibition [28]. A previous study reported that extract-induced NO inhibition could involve direct scavenging, via inhibition of iNOs activity or gene expression [31].

**Fig 3:**
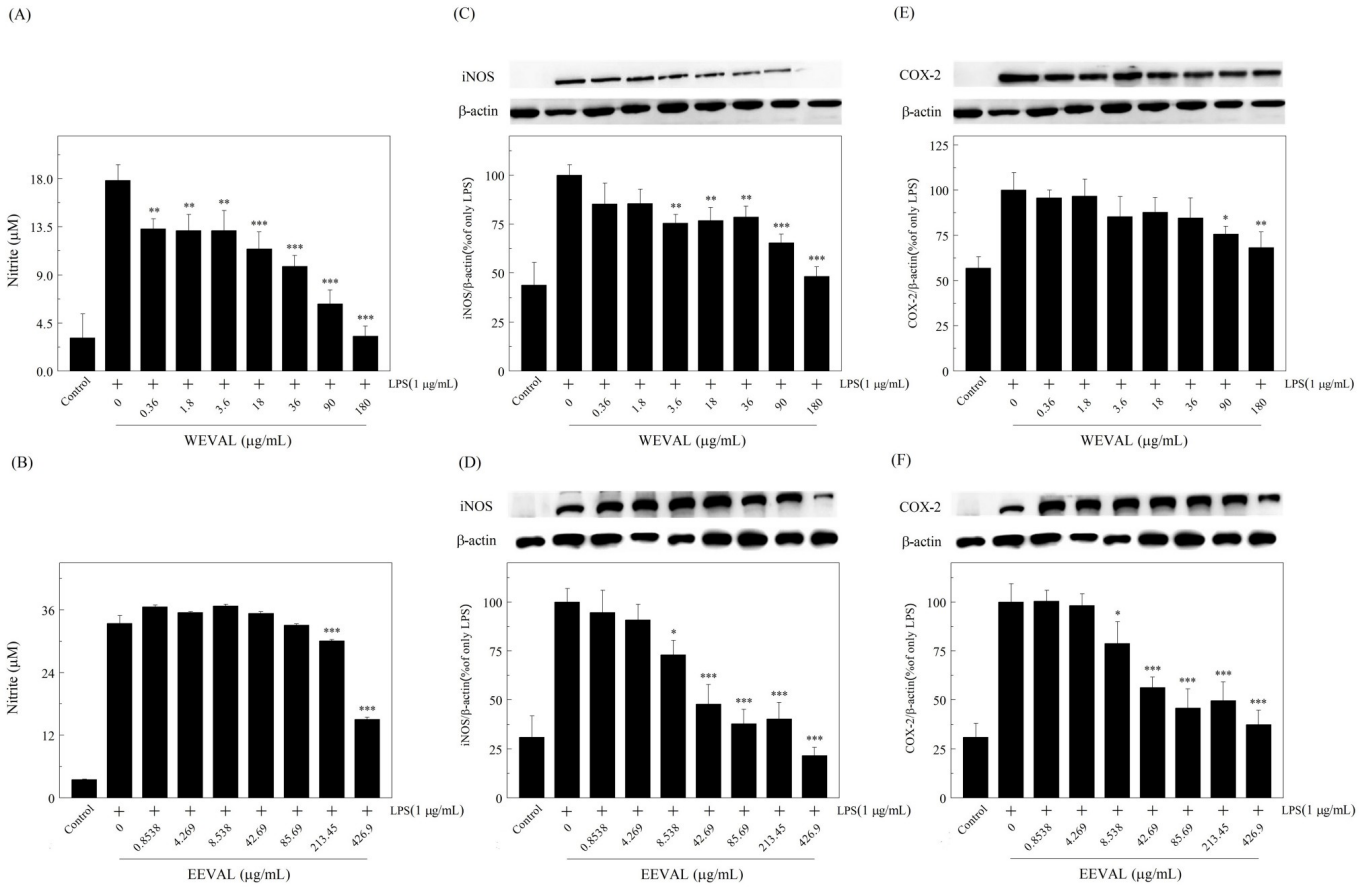
The effects of VA extracts on nitrite production, iNOS and COX-2 protein expression. The effects of (A) WEVAL and (B) EEVAL on nitrite production. The effects of (C) WEVAL and (D) EEVAL on iNOS protein expression and (E) WEVAL and (F) EEVAL on COX-2 protein expression. Results are expressed as mean ± SD of triplicate experiments. Statistically significant changes were clustered as significant (*) where p < 0.05, more significant (**) where p < 0.01 and highly significant (***) where p < 0.001 as compared with LPS-stimulated group.

We evaluated the effects of the VA leaf extracts on the gene expression of iNOs and NO production and found that both WEVAL and EEVAL showed dose-dependent inhibition of LPS-induced NO production in RAW 264.7 macrophage cells (Fig 3(A) and 3(B)) with inducible NOs production showing a similar trend (Fig 3(C) and 3(D)). These results indicated that the inhibition of NO production might have resulted from the inhibition of iNOs protein expression.

### 3.7. WEVAL and EEVAL inhibited COX-II

COX converts arachnoid acid to prostaglandin and thromboxane, which are both involved in inflammation. COX-II is primarily presented at the inflammation site where pro-inflammatory factors such as IL-1β and iNOS stimulate COX-II expression [32]. We measured COX-II reduction, which indicates inflammation attenuation, in the present and absence of VA leaf extracts. Fig 3(E) and 3(F) shows the effects of WEVAL and EEVAL on COX-II. The COX-II reduction rations of WEVAL at 180 μg/mL and EEVAL at 426.9 μg/mL were 31.7% and 62.6%, respectively. Similar to WEVAL and EEVAL inhibition of iNOS, we found a dose-dependent WEVAL and EEVAL inhibition of COX-II expression. EEVAL showed greater inhibition of COX-II production.

COX-II and iNOs are both important enzymes that mediate the inflammatory process. Inappropriate up-regulation of COX-II and iNOs gene expression could cause inflammatory diseases or even neoplastic disorders [33]. We found that the VA leaf extracts could decrease the levels of pro-inflammatory cytokines and the production of COX-II and iNOs in RAW 245.7 cells. Therefore, VA leaves appear to have both antioxidant and anti-inflammatory effects. Specifically, EEVAL was a more potent inflammation inhibitor and could offer potential medicinal uses.

### 3.8. WEVAL and EEVAL inhibited NF-kB translocation and MAPK phosphorylation

We assessed the levels of nuclear p65 and phosphorylated p38 to investigate whether VA leaf extracts could regulate the anti-inflammatory process via the NF-κB and MAPK.

NF-κB plays a critical role in immune and inflammation regulation. Upon inflammatory stimulation with lipopolysaccharide (LPS), TNF, irradiation or viral infection results in the activation of Toll-like receptor 4 and downstream inhibitor κB kinases (IKKs) NF-κB is released after phosphorylation and degradation of its inhibitory proteins; specifically, IκBs, and is transported from the plasma to the nucleus where it regulate transcription of its target genes[34]. We assessed the levels of the p65, which is an important trans-activating NF-κB domain, in the nucleus to determine NF-κB activation. Activated NF-κB induces the mRNA expression of pro-inflammatory cytokines, including TNF-α, IL-1β, and IL-6, to defend against infection lesions [25]. Non-steroidal anti-inflammatory drugs (NSAIDs) like aspirin controls the gene expression of cyclooxygenase (COX)-2 by suppressing NF-κB[35]. Moreover, decreased expression of phosphorylated IKKβ, IκBα, and NF-κB/p65 reduce NO production and iNOs mRNA expression [36].

MAPK constitute an important inflammatory signal cascade from the cell surface to the nucleus. LPS interacts with Toll-like receptor 4 and activates various MAPK pathways: ERK, JNK and p38, which mediated activation of pro-inflammatory transcription factors[37]. The NF-κB signaling pathway activation is closely linked to the activated MAPKs, which promote downstream transcription factors that increase inflammatory gene expression[38]. Although the molecular mechanism of NF-κB regulation through p38 MAPK(p38) remains elusive, p38 has been identified as an upstream regulatory NF-κB kinase [39]. Inhibition of the p38 attenuates NF-κB transcriptional activity; however, it does not affect NF-κB translocation to the nucleus and DNA binding [40]. Consequently, p38 blocking reduces the expression of NF-κB-mediated genes for pro-inflammatory factors, including TNF-α, IL-1β, COX-II, and iNOS[41, 42].

**Fig 4:**
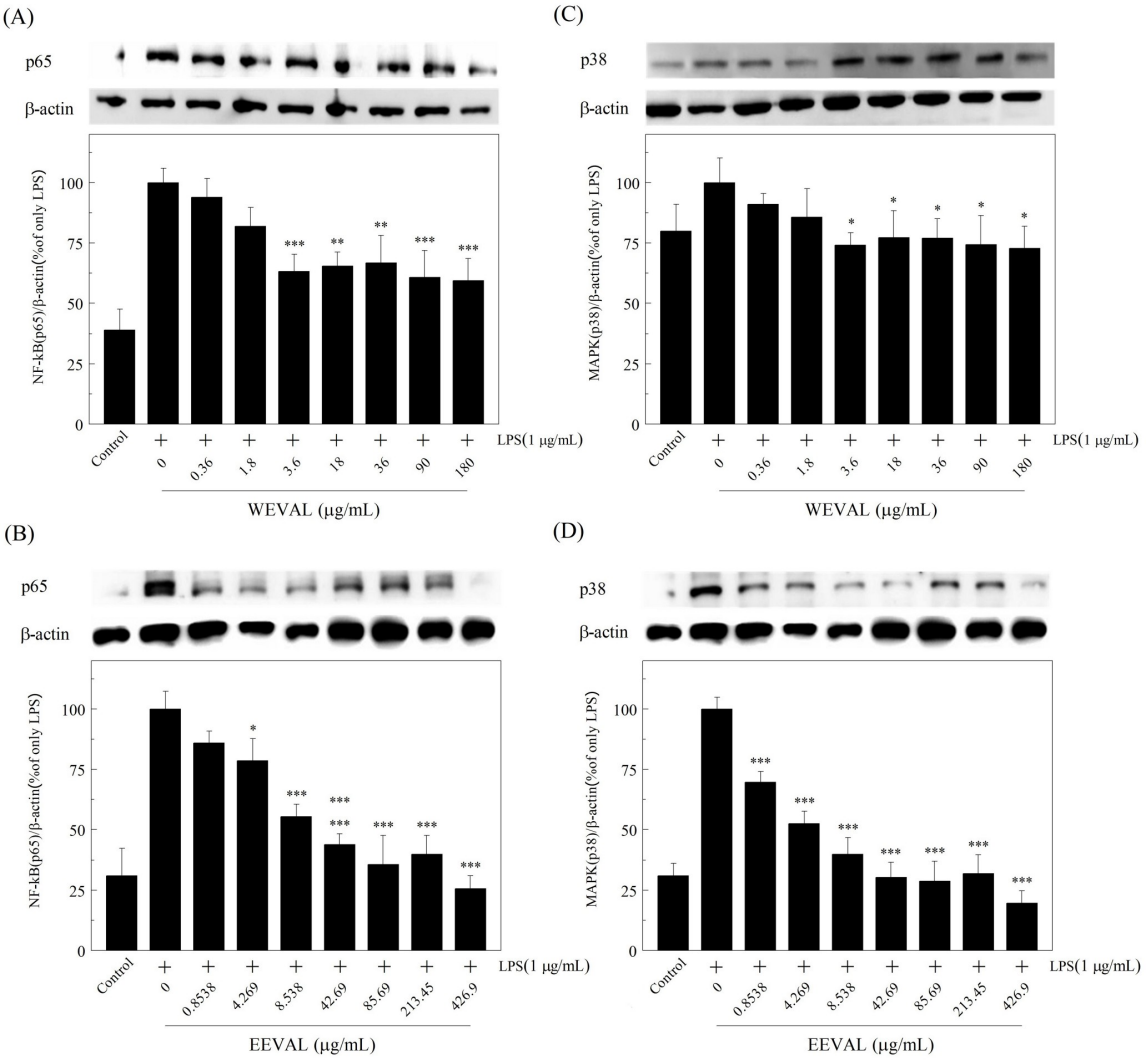
The effects of VA extracts on p65 and p38 protein expression in LPS-stimulated RAW 264.7 macrophages. The effects of (A) WEVAL and (B) EEVAL on p65 protein expression and the effects of (C) WEVAL and (D)EEVAL on p38 protein expression. Results are expressed as mean ± SD of triplicate experiments. Statistically significant changes were clustered as significant (*) where p < 0.05, more significant (**) where p < 0.01 and highly significant (***) where p < 0.001 as compared with LPS-stimulated group.

**Fig 5:**
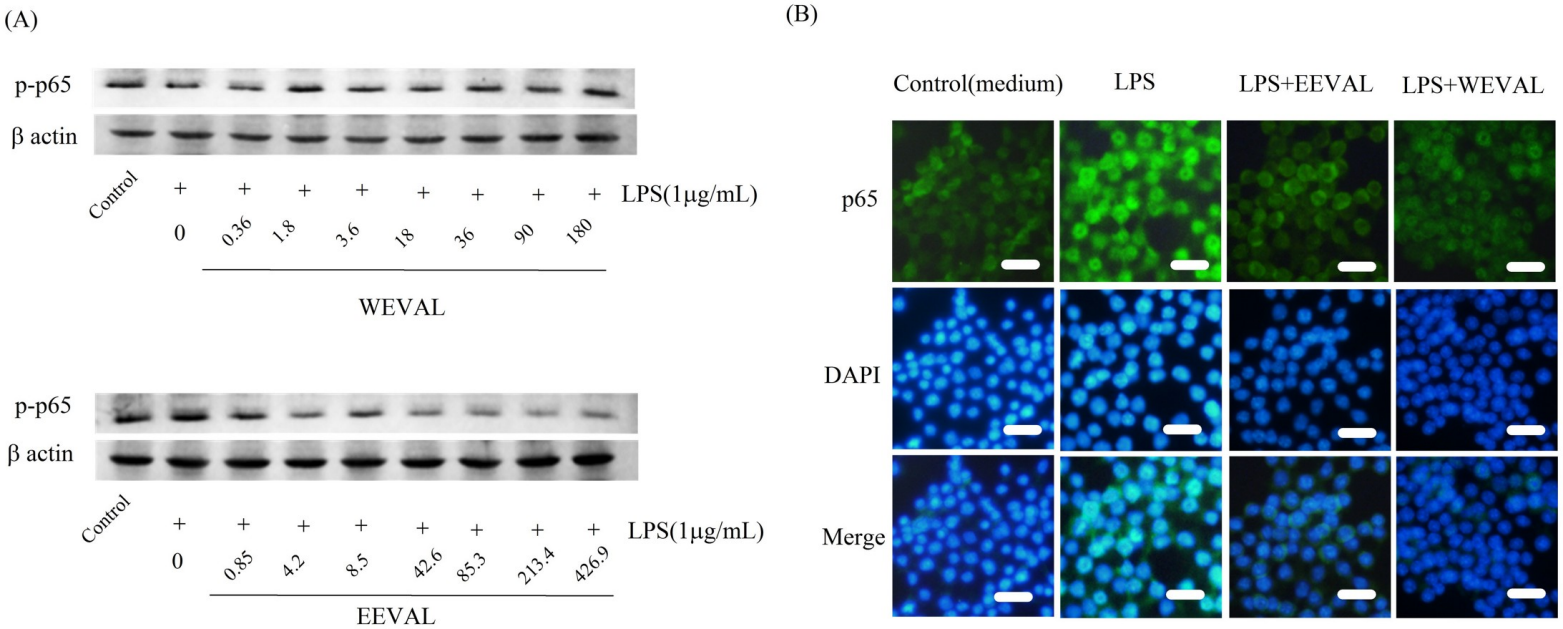
NF-ĸB p65 nuclear translocation in LPS-stimulated RAW 264.7 macrophages. (A) Effect of WEVAL and EEVAL on expression of phospho-NF-κB p65 was measured by western blot analysis. (B) Effect of WEVAL and EEVAL on NF-ĸB p65 protein translocation from cytosol to nucleus in macrophages. Nuclei were stained by DAPI. p65 expression was analyzed by immunofluorescence staining.

As shown in Fig 4(A) and 4(B), both WEVAL and EEVAL decreased total p65 levels in the LPS- stimulated RAW 264.7 cells. In Fig 5(A), the amounts of phospho-NF-κB p65 in the nuclei were decreased significantly after WEVAL and EEVAL treatments. In Fig 5(B), the immunofluorescense of phospho-NF-κB p65 indicated that WEVAL and EEVAL lessened the translocation of NF-κB p65 protein significantly. Further, WEVAL and EEVAL reduced the levels of phosphorylated p38 (Fig 4(C) and 4(D)).

**Fig 6:**
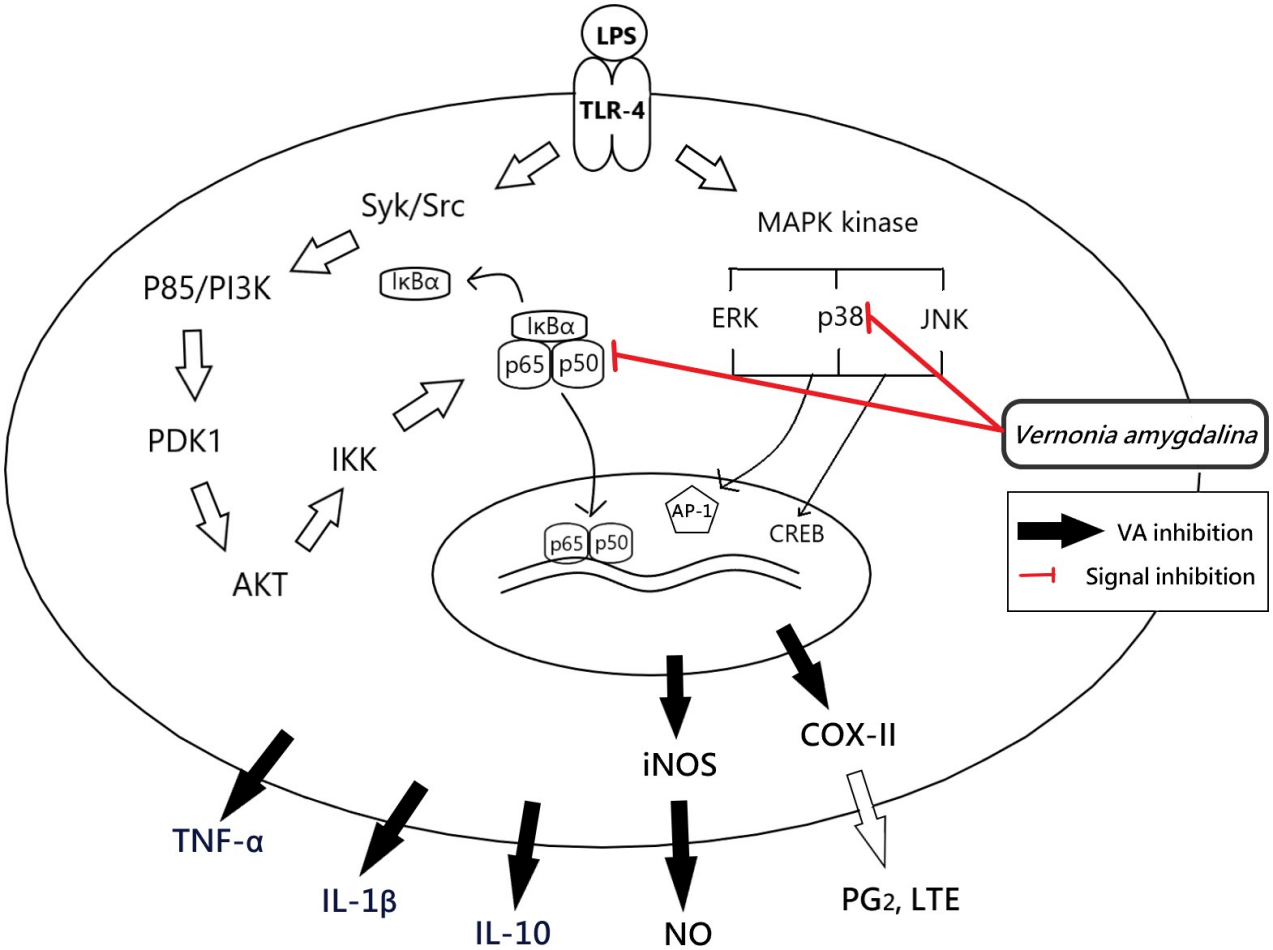
Mechanism of the anti-inflammation effect of *Vernonia amygdalina*.

Taking together, the results of VA anti-inflammatory effect were similar with previous plants extract reports such as *Syzygium cumini* seed[43], *Perilla frutescens* leaf [44], *Anemarrhena asphodeloides* rhizomes [45], which indicates that the inhibitory effects of VA leaves extract on pro-inflammatory cytokines, iNOs, and COX-II were regulated through the p38 MAPK and NF-κB signal pathway (Fig 6).

## 4. Conclusion

Our findings indicate that VA leaf extracts have antioxidant, DNA protection and anti-inflammation effects. To our knowledge, this is the first study to report anti-inflammatory effect of the VA and to explore its mechanism and the involved signaling pathway. VA leaf extracts obtained using different extraction methods showed differences in their antioxidant and anti-inflammation effects.

In anti-inflammatory effect, VA extracts were potent inhibitors of LPS-induced TNF-α, IL-1β, IL-10, iNOs, and COX-II production in macrophages. The underlying mechanism involved MAPK and NF-κB inhibition, which elicited down-regulation of the gene transcription of these pro-inflammatory factors. However, we found that the antioxidant and anti-inflammation abilities different between WEVAL and EEVAL. WEVAL yielded higher levels of phenol and flavonoid compounds, which are highly associated with the antioxidant ability of plants. Contrastingly, EEVAL had a stronger anti-inflammatory ability. In contrast to the antioxidant ability, the anti-inflammatory ability was not associated with the phenol and flavonoid levels. These results demonstrated that production of WEVAL and EEVAL from VA leaves may provide a safe and efficacious source of pharmaceutical applications, with antioxidant, DNA protective and anti-inflammation activities.

## Supporting information

S1 File (RAR)
